# Hypoxia-challenged MSC-derived exosomes deliver miR-210 to attenuate post-infarction cardiac apoptosis

**DOI:** 10.1186/s13287-020-01737-0

**Published:** 2020-06-08

**Authors:** Hao Cheng, Shufu Chang, Rende Xu, Lu Chen, Xiaoyue Song, Jian Wu, Juying Qian, Yunzeng Zou, Jianying Ma

**Affiliations:** 1 Department of Cardiology, Zhongshan Hospital, Fudan University, 1609 Xietu Road, Shanghai, 200032 China; 2 Shanghai Institute of Cardiovascular Diseases, Zhongshan Hospital and Institute of Biomedical Sciences, Fudan University, 180 Feng Lin Road, Shanghai, 20032 China

**Keywords:** Myocardial infarction, Exosome, Mesenchymal stem cells, Hypoxia, Apoptosis

## Abstract

**Background:**

Myocardial infarction (MI) is a major cause of death worldwide. Although percutaneous coronary intervention and coronary artery bypass grafting can prolong life, cardiac damage persists. In particular, cardiomyocytes have no regenerative capacity. Mesenchymal stem cells (MSCs) are attractive candidates for the treatment of MI. The manner by which MSCs exert a beneficial effect upon injured cells is a source of continued study.

**Methods:**

After the isolation and identification of exosomes from MSCs, the expression of miR-210 was determined by microarray chip. Subsequently, gain- and loss-function approaches were conducted to detect the role of exosomes and exosomal-miR-210 in cell proliferation and apoptosis of cardiomyocytes, as well as the MI in vivo. Dual-Luciferase Report Gene System was used to demonstrate the target gene of miR-210.

**Results:**

We tested the hypothesis that MSC-derived exosomes transfer specific miRNA to protect cardiomyocytes from apoptotic cell death. Interestingly, direct cardiac injection of MSC exosomes reduced infarct size and improved heart function after coronary ligation. In vitro, the MSC exosomes enhanced cardiomyocyte survival to hypoxia. Confirmation of exosome uptake in myocytes was confirmed. Dual-luciferase reporter assay implicated miR-210 as a mediator of the therapeutic effect and AIFM3 as a downstream target. Treatment with miR-210 overexpressing MSC exosomes improved myocyte protection to both in vitro and in vivo stress. Furthermore, the endogenous and exogenous miR-210 had the same therapeutic effects.

**Conclusion:**

These results demonstrated that the beneficial effects offered by MSC-exosomes transplantation after MI are at least partially because of excreted exosome containing mainly miR-210.

**Graphical abstract:**

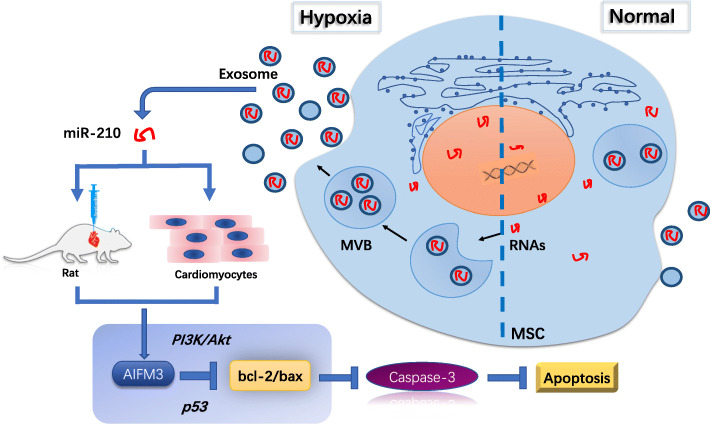

## Background

Ischemic heart disease is a significant cause of morbidity and mortality [1]. Percutaneous coronary thrombolytic therapy improves clinical outcomes. However, nearly 25% of patients are not re-perfused in a timely manner [2]. Furthermore, cardiomyocytes have little to no regenerative capacity. Cell-based therapies, such as stem cells, which have regenerative potential, are being applied to individuals with cardiac disease [3]. Stem cells can secret quantities of growth, anti-apoptotic, and anti-inflammatory factors that may improve heart function. However, technical issues have shown stem cells themselves to be inefficient [4]. Mesenchymal stem cells (MSCs) are prototypic adult stem cells [5] that produce paracrine growth factors that may increase survivability of cardiomyocytes and stimulate angiogenesis [6].

All cell types secrete membrane vesicles, including exosomes, that contain bioactive substances such as proteins, mRNAs, and miRNAs, and enzymes, among others [7]. During uptake by neighboring cells, exosome-delivered cargo has the potential to alter cell response [8]. Embryonic stem cell exosomes were reported to drive cardiac regeneration [9]. Others noted exosomes provided remote pre-conditioning to limit kidney injury [10]. Since prior studies have noted that MSC exosomes are cardio-protective, they are being tested as an off-the-shelf therapy for MI [11].

At the same time, MSC exosome treatment can alter miRNA levels [7]. miRNAs activated pro-survival kinases and induced a glycolytic switch to increase cellular resistance to hypoxic stress [12]. Post-MI individuals were found to have increased serum and decreased infarct tissue levels of miR-1 and miR-133a [13]. Parenthetically, miR-210 and miR-744 were found increased in exosomes in response to hypoxia. However, there was no miR-744 gene of rat in NCBI Gene. We chose miR-210 as our key role in myocardial protection. Furthermore, a number of studies have linked exosomal-miR-210 to protection from ischemic injury [14–16]. We hypothesized that MSCs secrete miRNA-210-enriched exosomes that, in a paracrine manner, protect cells and organs from injury. Herein, we show that exosome miR-210 limits hypoxia-driven myocyte apoptosis and tissue death following coronary ligation.

## Methods

### Animals

Animal experiments were approved by the Ethics Committee of Fudan University (reference number: 20140226-095). Wild type, male SD rats aged 10–12 weeks and newborn male SD rats were purchased from Shanghai Sippr-BK Laboratory Animal Co. Ltd. (Shanghai, China) and maintained under pathogen-free conditions.

### Rat model of myocardium infarction and assessment of heart functions

Male SD rats (~ 250 g) were anesthetized with 10% chloral hydrate (w/v; Acros, Japan) by intraperitoneal injection. The heart was exposed and the left anterior descending artery ligated. Sham-ligated rats served as controls. Animals underwent injection of the cardiac muscle close to the area of arterial ligation with the following agents: PBS, exosomes derived from MSCs, exosomes derived from MSCs cultured in 10 μM GW4869 that could inhibit exosome production sufficiently without toxic effects on cells [17], exosomes derived from MSCs infected with anti-miR-210, and exosomes from MSCs infected with empty lentivirus. The exosomes acquired from 1 × 10^6^ MSCs were delivered in 20 μl of PBS. Echocardiography was performed on days 0 and 28 after coronary ligation using a Vevo 2100 system (VisualSonics Inc., Toronto, ON, Canada) with an 80-MHz probe. Upon study completion, animals were euthanized, and tissues were harvested.

### Cell isolation and culture

Bone mesenchymal stem cells were isolated from the femurs of SD rats and seeded onto culture dishes. Then they were cultured in DMEM/F12 supplemented with 10% fetal bovine serum (FBS; Invitrogen, Carlsbad, CA, USA) at 37 °C and 5% CO_2_ until confluent. Medium was replaced every 2 days. At passage 3, the phenotype of MSCs was confirmed by flow cytometry using antibodies against rat CD90-APC and CD45-PEcy7 (#553080, BD Bioscience, San Diego, CA, USA). The SD Rat MSC Osteogenic Differentiation Basal Medium and Adipogenic Differentiation Basal Medium (Cyagen Bioscience, Guangzhou, China) were used to promote the differentiation of MSCs to further confirm differentiation capacity.

### Exosome isolation

Exosomes from culture supernatants were isolated by ultracentrifugation. Collected cell culture supernatant was subjected to several centrifugations (300, 2000, and 10,000*g* for 15, 15, and 40 min, respectively). After each centrifugation, the supernatant was filtered through 0.22 μm filters and the resultant was collected. Then the resultant was subjected to centrifugation at 110,000*g* for 75 min to yield a pellet that was suspended in PBS and then centrifuged again at 110,000*g* for 75 min. The pellet obtained with the final centrifugation was considered the exosomes.

A BCA assay kit (Beyotime, China) was used to analyze the protein level of lysed exosomes (50μl RIPA lysis buffer, Beyotime, China). CD63 and TSG101 protein levels were detected by Western blot. A mirVana miRNA isolation kit (Invitrogen, Austin, TX, USA) was used to isolate exosome miRNA, and relative expression levels of miR-210 were determined by q-PCR.

### Transmission electron microscopy

For electron microscopy analysis, exosome suspensions were absorbed onto formvar carbon-coated EM grids. Three grids were prepared for each exosome sample. An absorbing page was used to gently remove excess liquid. Then, the exosome suspension was subjected to 2.5% uranyl acetate staining for 7 min. Grids were washed three times with PBS and maintained in a semi-dry state. Samples were observed using a Hitachi-8100IV transmission electron microscope (Hitachi, Tokyo, Japan) at 100 kV.

### Quantitative real-time PCR analysis

Total RNA was extracted from cells using TRIzol reagent (Invitrogen, Austin, TX, USA) following the manufacturer’s instructions. Reverse-transcript reactions were conducted using the PrimeScript RT reagent kit (Takara, Japan). qPCR primers were purchased from Tiangen Biotech Co. Ltd. (Beijing, China). The has-miR-210 primers were CTGTGCGTGTGACAGCGGCTGA. qPCR was conducted using a standard SYBR Green PCR kit (Toyobo, Osaka, Japan) protocol on an Applied Biosystems 7500 Real-Time PCR System (Applied Biosystems, Foster City, CA, USA). The relative mRNA expression level was analyzed by the 2^(−∆∆CT)^ method.

### Co-culture of cardiomyocytes and exosomes

Cardiomyocytes were isolated from newborn male SD rats with 1 mg/mL collagenase II (Invitrogen, Austin, TX, USA). After 3 days, the isolated cardiomyocytes were co-cultured with exosomes derived from MSCs, MSCs treated with GW4869, and MSCs transfected with miR-210 agomir, miR-210 antagomir, or negative vehicle. After 48 h, cardiomyocytes were collected for subsequent analyses.

### Viability assay

Cell viability was evaluated by LDH-release assay (Beyotime, China) and CCK8 assay (Beyotime, China). Cardiomyocytes in 6-well plates were challenged with hypoxia ± the indicated treatment. Culture supernatants were aliqouted to fresh 96-well plates with LDH-release assay buffer. Absorbance at 492 nm and 630 nm was measured with a Multi-Mode Microplate Reader (BioTek, Winooski, VT, USA) controlling for background signal. In other experiments, cells were treated as above and CCK8 reagent was added and absorbance at 450 nm measured.

### Colocalization of miR210 and exosomes

Rat BMSCs P3 generation cells in good condition were digested with trypsin then centrifuged. The cells were resuspended in complete medium and were spread in 4 wells of 6-well plate. The cell density will reach 80% next day. The cells were transfected with miR210 mimics. The transfection systems were (a) 125 μl Opti-MEM + 7 μl Lipofectamine3000; (b) 125 μl Opti-MEM + 50 nM miR-210 mimics + 10 μl P3000 Reagent. Twenty-four after transfection, the medium was replaced with low-glucose DMEM without FBS. The cells in 2 wells were placed in a 5% CO_2_ incubator at 37 °C for 24 h; the cells in the other 2 wells were placed in an anoxic incubator for 24 h. The next day, the exosomes were isolated from the cell culture medium by ultracentrifugation. Then the exosomes were incubated with CD63 (1:100) and CD81 (1:100) at 4 °C overnight. After 24 h, the secondary antibodies were added and incubated in room temperature for 1 h in the dark. The secondary antibody corresponding to CD63 was goat anti-rabbit (1;100), blue fluorescence (Alexa Fluor 350); the secondary antibody corresponding to CD81 was donkey anti-mouse(1;100), red fluorescence (Alexa Fluor 555); and miR210 mimics come with green fluorescence (5’FAM). Finally, we used laser confocal microscope to observe and take pictures.

### Exosomes endocytosis into cardiomyocytes

Rat Bone MSCs (BMSCs) P3 generation cells in good condition, were digested with trypsin then centrifuged. The cells were resuspended in complete medium and were spread in 2 wells of 6-well plate. The cell density will reach 80% the next day. The cells were transfected with miR210 mimics. The transfection systems and exosomes isolation procedures have been mentioned above. The exosomes were resuspended with high-glucose DMEM to make conditioned medium for cardiomyocytes. The cardiomyocytes were divided into 0 and 24 h culture groups. After reaching the time, the cell slides were removed and rinsed twice with PBS. It was treated with 4% paraformaldehyde under room temperature for 10 min and then rinsed with PBS three times, each 5 min. Then the 0.5% Txiton X-100 was added for 5 min under room temperature and rinsed with TBST three times, each 5 min. Next, the DAPI was treated for 10 min away from light and then rinsed with PBS three times, each 5 min. Finally, we used mount slides and took pictures under laser confocal microscope.

### Dual-luciferase reporter assay

In order to construct the overexpression vector PGL3-AIFM3 promoter region, PCR primers were designed and synthesized based on the sequence information of the AIFM3 (NM_001013977.3) promoter region in the NCBI (see Supplemental Table 1). The AIFM3 promoter region was used for the PCR target gene, plus Kpn I/Xho I restriction sites at the 5′ UTR of the AIFM3 promoter region-F and AIFM3 promoter region-R primers to ligate to the vector PGL3. For analysis of luciferase activity, human embryonic kidney cells (HEK293T, ATCC) were cultured in 6-well plates and transfected. The transfection systems were (a) 150 μl Opti-MEM + 4 μl Lipofectamine2000 and (b) 150 μl Opti-MEM + 3 μl plasmid/50 nM miR-210/0.3 μl RL-TK. The four groups were PGL3 + RL-TK, miR-210 + PGL3 + RL-TK, miR-210 + PGL3-AIFM3 + RL-TK, and miR-210 control + PGL3-AIFM3 + RL-TK. After growing 24 h, the cells were collected for application in the Dual-Luciferase Reporter Assay System (Beytime) using a GloMax 20/20 Luminometer (Promega, Winooski) under recommended condition. After lysed for 15 min at room temperature, renilla luciferase activity was employed as internal control, and the ratios of firefly luciferase luminescence relative to control were measured.

### Western blot

All proteins from cells or myocardial tissue were extracted and quantified. Micro BCA™ Protein Assay Kits (Thermo Fisher Scientific, Waltham, MA) were used to quantify protein levels. Equivalent amounts of protein was electrophoresed through 12% SDS-PAGE (stacking gel, 70 V; separating gel, 110 V) and transferred to nitrocellulose membranes (200 mA, 50 min). Blots were incubated for 1 h at room temperature with the indicated antibodies (CD63: ab108950, 1:1000, Abcam, CA, USA; TSG101: ab125011, 1:5000, Abcam; Cleaved-Caspase-3: ab2302, 1:500, Abcam; Bcl-2: ab 196,495, 1:1000, Abcam; Bad: ab32445, 1:5000, Abcam; Bax: ab32503, 1:5000, Abcam), and then incubated with goat-anti-rabbit/mouse HRP-linked secondary antibody (Abcam, Cambridge, MA, USA). Chemiluminescence substrate (Pierce Chemical, Rockford, IL, USA) was used to visualize protein signals. The intensity of the protein bands was analyzed by ImageJ software (NIH, USA).

### Statistical analysis

All experiments were repeated at least three times. Data is shown as the mean ± standard error. Statistical analysis was carried out using GraphPad Prism software (version 5.01; San Diego, CA, USA). The unpaired *t* test was used to compare data between groups. A *P* value < 0.05 was regarded as statistically significant.

## Results

### Preparation and characterization of hypoxia-challenged exosomes

**Fig. 1:**
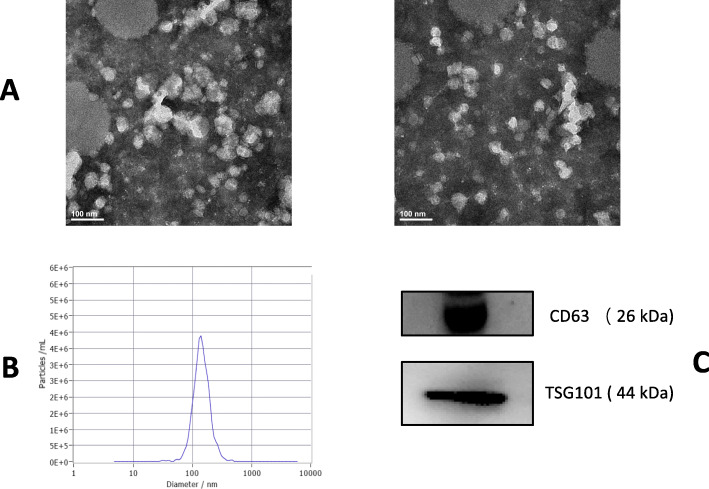
Characterization of exosomes derived from MSCs. **a** Transmission electron micrographs of exosomes. Scale bar, 100 nm. Arrows, representative individual exosomes. **b** Nanoparticle Tracking Analysis(NTA) of exosomes. **c** Western blot analysis of CD63 and TSG101 expression in exosomes. Each experiment was repeated 3 times

Rat bone-derived MSCs were cultured for 72 h under hypoxia (1% O_2_). Exosomes were isolated from MSC-conditioned medium and characterized by transmission electron micrography (TEM), Nanosight Tracking Analysis (NTA), and expression of the exosome surface markers (Fig. 1a–c). TEM showed that MSC-derived exosomes displayed a characteristic cup-shaped structure. NTA demonstrated exosome sizes ranged from 30 to 150 nm. Finally, Western blotting analysis of lysed exosomes detected the exosome markers, CD63 and TSG101. Furthermore, the secretion of exosome in the supernatant of MSCs cultured under hypoxia was significantly increased compared with that in normoxic culture (Supplemental Fig. 1A).

### MSC exosomes protect against cardiomyocyte death

**Fig. 2:**
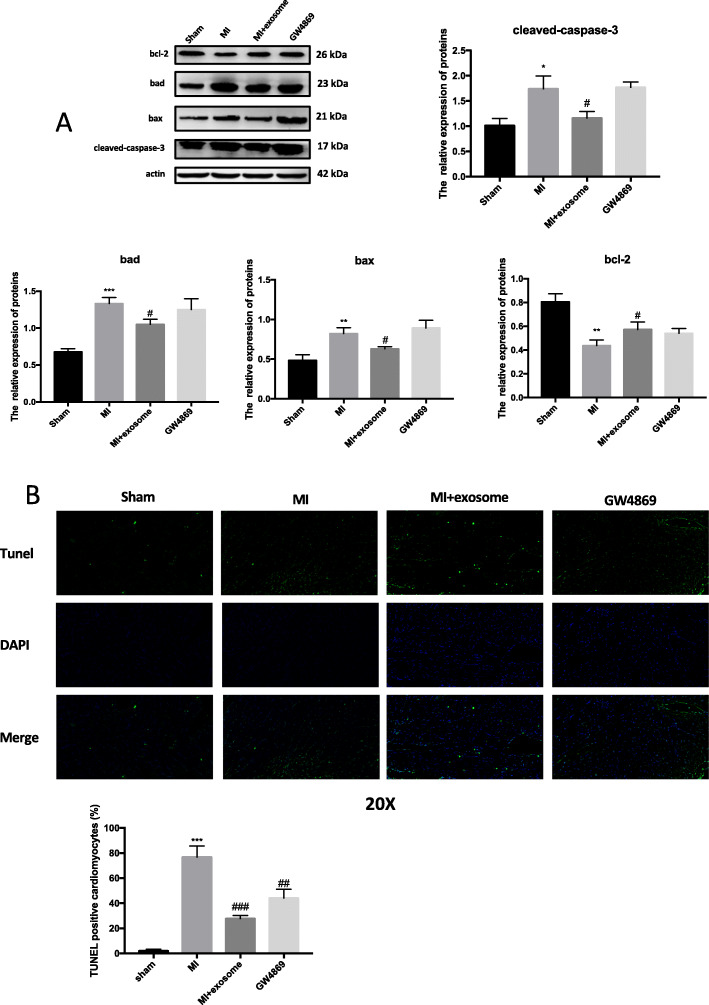
Exosomes derived from MSCs inhibit apoptosis after myocardial infarction. **a** Expression levels of the apoptotic proteins BAD, BAX, BCL-2, and Cleaved-CASPASE-3 were evaluated in cardiac tissue sections from sham-operated rats and coronary-ligated rats that received either no therapy (PBS), MSC exosomes, or exosomes from MSCs treated after vessel occlusion with GW4869 immediately (*N* = 5 per group). Western blot analysis of protein lysates obtained from the infarct border zone. Quantitative analysis of BAD, BAX, BCL-2, and Cleaved-CASPASE-3 is shown in the lower panel. **b** Cell injury was determined by TUNEL assay. Fluorescence staining with vital dyes shown in blue indicates live cardiomyocytes, whereas ethidium homodimer-1 staining labels dead cells green. The percentage of cardiomyocytes death is shown in the right panels. Each experiment was repeated 3 times. *p < 0.05, **p < 0.01, ***p < 0.001 for MI vs. sham. ^#^*p* < 0.05 for MI + exosome vs. MI

To test the potential of MSC exosomes to provide cardio-protection, we established a rodent model of MI. Upon coronary ligation, animals received the described treatments directly to the ischemic myocardium. Seven days post-injury, animals were euthanized and heart tissues were assessed for protein expression of several apoptosis-related genes (Cleaved-CASPASE-3, BAD, BAX, BCL-2). Cleaved-CASPASE-3 and BAX were increased in tissue samples from post-MI animals compared to sham-operated controls. Post-MI animals treated with MSC exosomes showed a decrease in levels of Cleaved-CASPASE-3, BAD, and BAX compared to controls. However, exosomes from MSCs cultured with GW4869, which limits exosome formation [17], did not decrease MI-related elevations in apoptosis proteins (Fig. 2a), suggesting a dose-dependent effect. Immunohistochemistry confirmed a significant decrease in the number of apoptotic cardiomyocytes in tissue samples from animals treated with exosomes versus those given MSCs-GW4869-exosomes or PBS (Fig. 2b).

### MSC exosomes increase cardiomyocyte viability

**Fig. 3:**
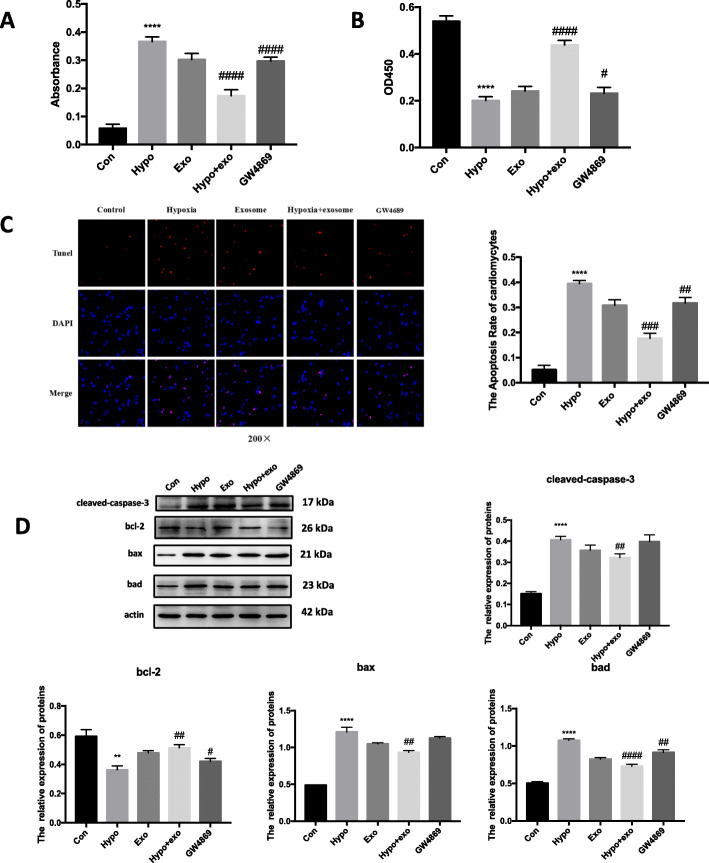
Exosomes derived from MSCs reduce cell death. Hypoxic cardiomyocytes cultured in 1% O_2_ for 24 h and treated with the indicate agents. Cells were divided into five groups: control group (cardiomyocytes were cultured in 21% O_2_), hypoxia group (cardiomyocytes were cultured in 1% O_2_), exosome group (exosomes were derived from MSC cultured in 21% O_2_, and cardiomyocytes were cultured in 1% O_2_), hypoxia + exosome group (exosomes were derived from MSC cultured in 1% O_2_, and cardiomyocytes were cultured in 1% O_2_), and GW4869 (MSCs were firstly treated with 10 μM GW4869, then exosomes were derived the MSCs and cardiomyocytes were cultured in 1% O_2_). Cell injury was determined by LDH release assay (**a**), MTT assay (**b**) and TUNEL assay (**c**). Fluorescence staining with vital dyes shown in green indicates live cardiomyocytes, whereas ethidium homodimer-1 staining labels dead cells red. The percentage of cardiomyocytes death is shown in the right panels. **d** BAD, BAX, BCL-2, and Cleaved-CASPASE-3 levels were evaluated by Western blot and expression quantified. Each experiment was repeated 3 times. **p < 0.01, ****p < 0.0001 for Hypo vs. Con. ^####^*p* < 0.0001 for hypo + exo or GW4869 vs. hypo

Based on the above in vivo studies, we assessed the effects of MSC exosomes on cultured neonatal cardiomyocytes. MI is associated with profound cellular hypoxia. Therefore, we challenged cardiac neonatal myocytes with hypoxia and determined LDH levels as a maker of cell injury. Interestingly, hypoxic myocytes treated with exosomes released less LDH compared to hypoxia myocytes; this effect was lost in cells treated with exosomes from GW4869-exposed MSCs (Fig. 3a). Furthermore, MSC exosomes prevented the hypoxia-mediated decrease in myocyte viability. As expected, exosomes from GW4869-exposed MSCs did not limit the hypoxia-mediated decrease in myocyte viability (Fig. 3b). We hypothesized that hypoxia induced apoptosis in cultured myocytes. As seen in the micrographs, the number of TUNEL-positive myocytes was increased by hypoxia, which was abated when cells were co-treated with MSC exosomes (Fig. 3c). In this case, exosomes from GW4869-trated MSCs did not limit hypoxia-induced apoptosis (Fig. 3c). Similar to results in tissue samples from cardiac infarct regions, hypoxic cardiomyocytes showed increased protein expression of multiple apoptotic-associated genes as well as the programed cell death mediator, caspase 3. These changes were abrogated when hypoxic myocytes were treated with MSC exosomes (Fig. 3d).

### miR-210 is abundant in MSC exosomes and has protective effects

**Fig. 4:**
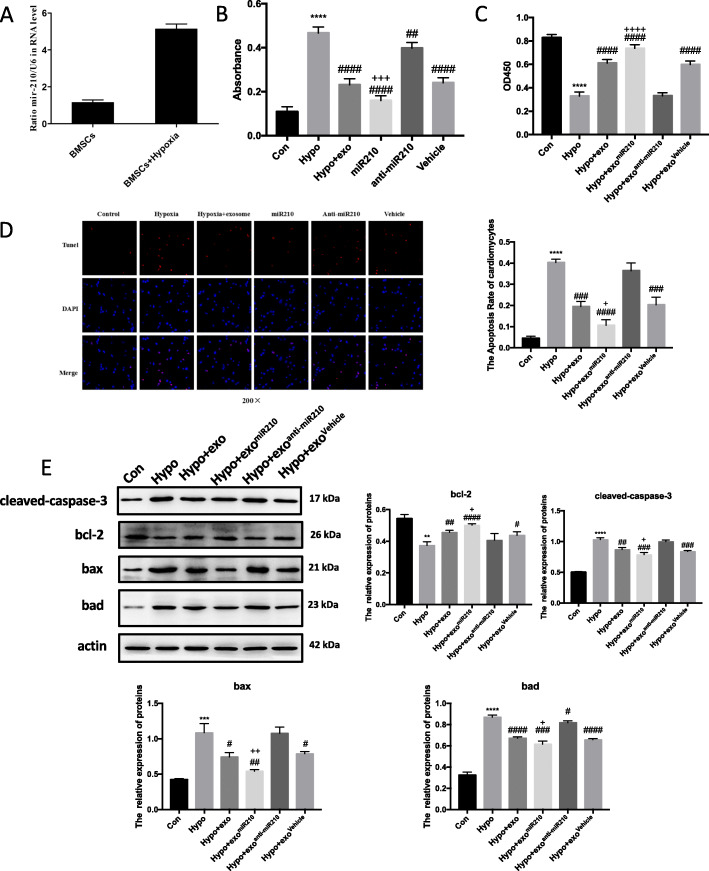
Overexpression of miR210 in MSC exosomes strengthens their cardioprotective function in vitro. **a** Polymerase chain reaction quantification of miR210 in the exosomes obtained from MSCs. Hypoxic cardiomyocytes were treated with exosomes obtained from MSCs that were pre-treated with miR210 (exo^miR210^), anti-miR210 (exo^anti-miR210^), or scramble (exo^Vehicle^) for the indicted time and cell injury characterized by LDH release (**b**), viability by MTT assay (**c**), and apoptotic programmed cell death by TUNEL assay (**d**). Red, TUNEL-positive nuclei; blue, DAPI-stained nuclei; green, troponin-positive cardiomyocytes. Scar bar × 200. The percentage of TUNEL-positive cells is shown in the right panels. **e** Western blot identification of BAD, BAX, BCL-2, and Cleaved-CASPASE-3 in cardiomyocyte lysates after cell incubation with the indicated exosomes ± hypoxia. Quantitative analysis of BAD, BAX, BCL-2, and Cleaved-CASPASE-3 is shown in the lower panel. Each experiment was repeated 3 times. **p < 0.01, ***p < 0.001, ****p < 0.0001 for hypo vs. con. ^#^*p* < 0.05, ^##^*p* < 0.01, ^###^*p* < 0.001, ^####^*p* < 0.0001 for hypo + exo, hypo + exo^miR210^, hypo + exo^anti-miR210^_,_ or hypo + exo^Vehicle^ vs. hypo. ^+^*p* < 0.05, ^++^*p* < 0.01, ^+++^*p* < 0.001, ^++++^*p* < 0.0001 for hypo + exo^miR210^ vs. hypo + exo

To identify the exosome cargoes possibly responsible for the therapeutic effects, we analyzed exosomal miRNAs. Microarray screening found miR-201 to be differently increased in exosomes from hypoxic MSCs compared to normoxic MSC-derived exosomes (Supplemental Fig. 1B). Microarray results were confirmed by q-PCR (Fig. 4a). To determine if miR-201 was sufficient or necessary to the therapeutic exosome effect, we pretreated MSCs with a miR-210 oligonucleotide (MSC-exo^miR-210^), anti-miR-210 oligonucleotide (MSC-exo^anti-miR-210^), or a scrambled control (MSC-exo^NC^) and harvested exosomes as described. Hypoxic myocytes treated with either regular MSC exosomes of MSC-exo^miR-210^ showed less LDH release and increased cell viability (on CCK8 assay) consistent with less injury (Fig. 4b). Conversely, cells treated with MSC-exo^anti-miR-210^ experienced increased cell injury and less viability (Fig. 4a, b). Similar results were observed on CCK-8 analysis and TUNEL assay among the cell treatment groups (Fig. 4c, d). Furthermore, BAX, BAD, and Cleaved-CASPASE 3 expression were higher in the hypoxia group compared to the MSC exosomes and MSC-exo^miR-210^ groups (Fig. 4e).

### MSC-secreted exosomal-miR-210 provides increased cellular survival against the hypoxia-induced myocardium injury

**Fig. 5:**
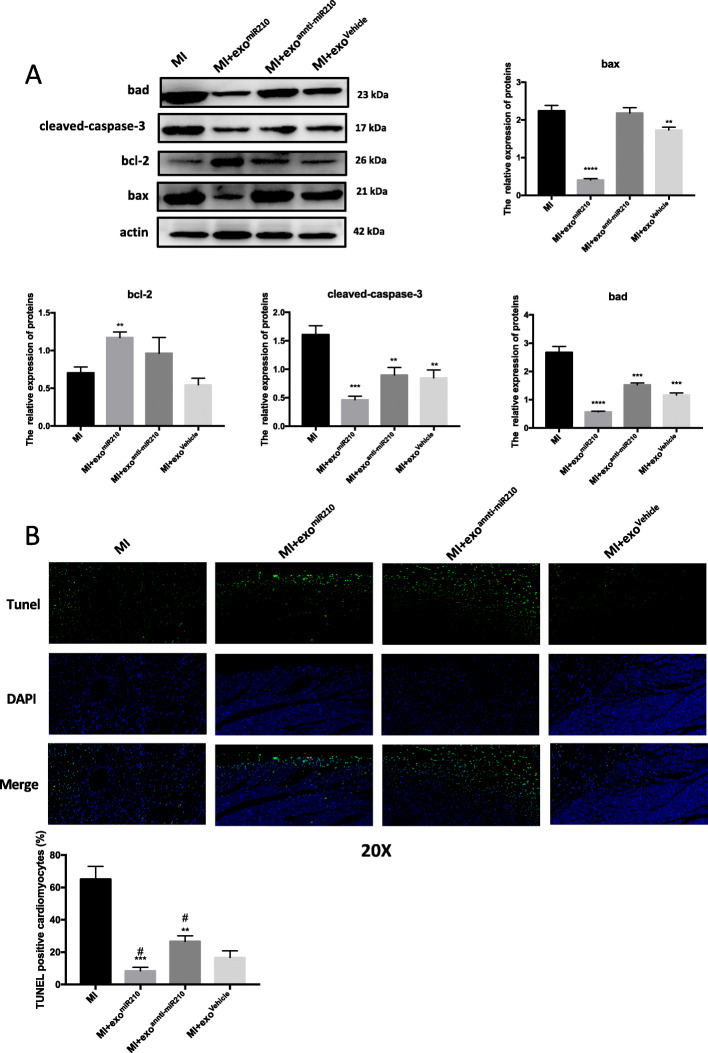
Exosomes derived from MSCs with miR-210 overexpression inhibit apoptosis after myocardial infarction. **a** Western blot analysis of protein levels of apoptosis-associated genes BAD, BAX, BCL-2, and Cleaved-CASPASE-3 from cardiac tissue samples from mice in the indicated treatment groups. Each experiment was repeated 3 times. **b** Cell death in tissue samples was characterized by TUNEL assay. **p < 0.01, ***p < 0.001, ****p < 0.0001 for MI + exo^miR210^, MI + exo^anti-miR210^, MI + exo^Vehicle^ vs. MI. ^#^*p* < 0.05 for MI + exo^miR210^, MI + exo^anti-miR210^ vs. MI + exo^Vehicle^

To validate the protective effects of miR-210 in vivo, we induced cardiac ischemia via coronary ligation and injected at risk myocardium with either PBS, MSC-exo^miR-210^, MSC-exo^anti-miR-210^, or MSC-exo^NC^. As detailed above, BAX, BAD, and Cleaved-CASPASE 3 levels were increased in tissue samples from the MI group. However, these effects were abolished when rats were treated with MSC-exo^miR-210^ (Fig. 5a). Conversely, treatment with MSC-exo^anti-miR-210^ did not alter injury-mediated increases in apoptotic-associated proteins (Fig. 5a). Apoptotic cells were increased in tissue samples from infarcted zones; this was less so in similar samples from animals treated with MSC-exo^miR-210^ (Fig. 5b).

### Exosomal-miR-210 improves heart function and reduces cardiac fibrosis following coronary ligation

**Fig. 6:**
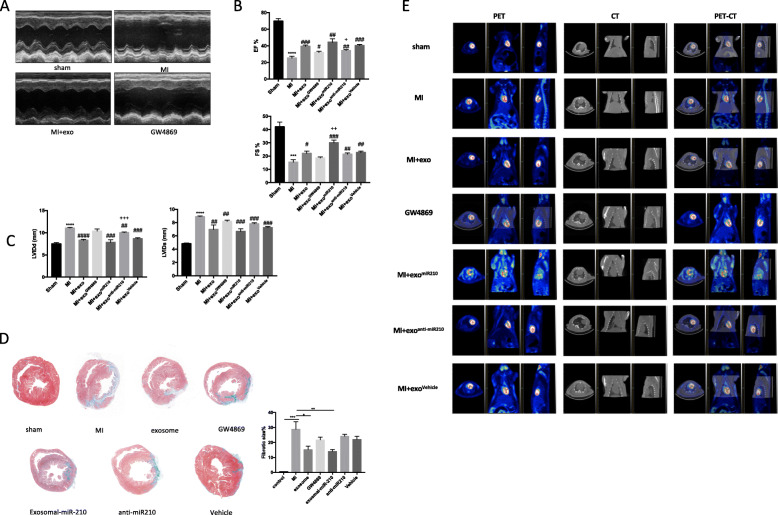
Overexpression of miR-210 in MSC exosomes leads to improvement of cardiac function and infarct size in vivo at 1 month after MI. **a**–**c** Representative photographs of M-mode echocardiography. Quantitative analysis of echocardiography (*N* = 4 per group). FS, fractional shortening; EF, ejection fraction; LVIDd, left ventricular internal diameter end-diastolic; LVIDs, left ventricular internal diameter end-systolic. **d** Masson Trichrome staining of whole heart tissue sections 28 days post-ligation and treatment with PBS, MSC exosomes, exosomes derived from MSCs treated with GW4869, MSCs-exo^miR210^, MSCs-exo^anti-miR210^, or MSCs-exo^Vehicle^. Percentage of tissue fibrosis is quantified and presented. *N* = 4 per group. **e** Representative photographs of PET-CT of rats from the respective treatment groups. *N* = 3 per group. ***p < 0.001, ****p < 0.0001 for MI vs. sham. ^#^*p* < 0.05, ^##^*p* < 0.01, ^###^*p* < 0.001, ^####^*p* < 0.0001 for MI + exosome, GW4869, MSCs-exo^miR210^, MSCs-exo^anti-miR210^, MSCs-exo^Vehicle^ vs. MI. ^+^*p* < 0.05, ^++^*p* < 0.01, ^+++^*p* < 0.001 for MSCs-exo^miR210^, MSCs-exo^anti-miR210^ vs. MSCs-exo^Vehicle^

To further evaluate the protective effects of miR-210, we used echocardiography and PET-CT to assess the left ventricular ejection and changes in ventricular remodeling 4 weeks after myocardial infarction. Echocardiography revealed that the MSC-exo^miR-210^ treatment group, similar to the sham surgery animals, had improved cardiac performance and less dysfunctional ventricular remodeling compared to the MI-PBS group and the MSC-exo^anti-miR-210^ group (Fig. 6a–c). Figure 6a shows that post-injury MSC-exo^miR-210^-treated rats had a significantly less decrease in ejection fraction (EF) (44.36% ± 3.34% vs. 25.21% ± 1.80%, *p* < 0.05) and less fractional shortening (FS) (30.03% ± 1.68% vs. 15.41% ± 1.53%, *p* < 0.05), with decreased left ventricular diastolic volume (LVIDd) (7.81 ±0.50 mm vs. 11.05 ± 0.13 mm, *p* < 0.001) and LVIDs (6.66±0.32 mm vs. 8.89± 0.09 mm, *p* < 0.001), compared with MI-PBS-treated rats. PET-CT showed that the cardiac protective effects were much better in the MSC-exo^miR-210^ group than in the MSC-exo group (Fig. 5e). Lastly, infarct-related tissue fibrosis (blue color on whole heart mounts) was significantly greater in the MSC-exo^anti-miR-210^ group than in rats treated with MSC-exo and MSC-exo^miR-210^ (Fig. 5d).

### miR-210 regulates PI3K/AKT and p53 signaling by targeting AIFM3

**Fig. 7:**
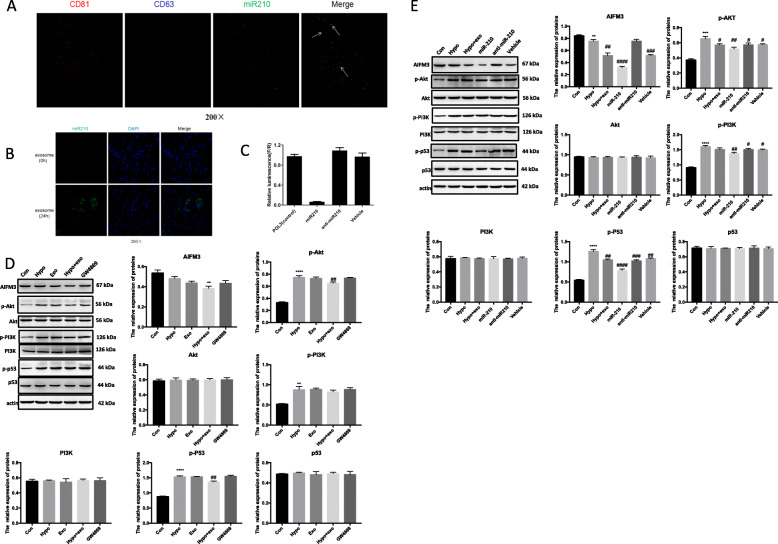
Hypoxia-mediated apoptosis is associated with changes in PI3K and AKT. **a** Co-localization of miR-210 with exosome-specific surface marker CD81 and CD63 by the immunofluorescence. **b** The uptake of exosome by cardiomyocytes, and the localization of miR-210 molecule with Cy3 fluorescence in cardiomyocytes. **c** Dual-luciferase reporter assay showed that AIFM3 was a target of miR-210. F, Firefly luciferase; R, Renilla luciferase. **d**, **e** AIFM3, p-AKT/AKT, p-PI3K/PI3K, and p-p53/p53 levels were evaluated by Western blotting in cardiomyocytes from the indicated treatment groups ± hypoxia. Quantifications are shown in the lower panel. *N* = 3 independent experiments. **p < 0.01, ****p < 0.0001 for hypo vs. con. ^##^*p* < 0.01 for hypo + exo vs. hypo

While exosomes, and especially miR-201 over-expressing exosomes, improved cell and organ responses to stress, it was not clear if miR-201 was actually delivered to myocytes. Based on immunofluorescence microscopy, we detected co-localization of miR-210 within exosomes (Fig. 7a). Further confirmation of exosome endocytosis was obtained using continuous live cell imaging of cardiomyocytes treated with conditioned medium enriched for MSC-exo^miR-210^ (Fig. 7b). miRNAs work at the post-transcriptional levels to regulate gene expression. According to the previous literatures and TargetScanHuman, an online prediction of microRNA targets (http://www.targetscan.org/), AIFM3 might be a target of miR-210 [18]. To test this hypothesis, dual-luciferase reporter assay was performed (Supplemental Fig. 2 and Table 1). Dual-luciferase reporter gene analysis of myocytes treated with MSC-exo^miR-210^ revealed that firefly luciferase activity was significantly inhibited when co-transfected with miR-210, indicating that AIFM3 as a down-stream target of miR-210 (Fig. 7c). We confirmed this result via Western blotting and q-PCR in miR-210 mimic-transfected cardiomyocytes. RNA levels of AIFM3 were significantly reduced in cardiomyocytes transfected with the miR-210 mimic (Fig. 7d). Studies have linked AIFM3/p53 and PI3K/Akt signaling pathways in the setting of MI [18]. Therefore, we investigated the crosstalk between these two signaling pathways. Western blotting analysis suggested that levels AIFM3 were upregulated and levels of p-AKT, p-PI3K, and p-p53 were downregulated (Fig. 7c). After transfection with the miR-210 mimic, similar results were obtained (Fig. 7d).

### The comparison of regulatory activity between endogenous and exogenous miR-210

**Fig. 8:**
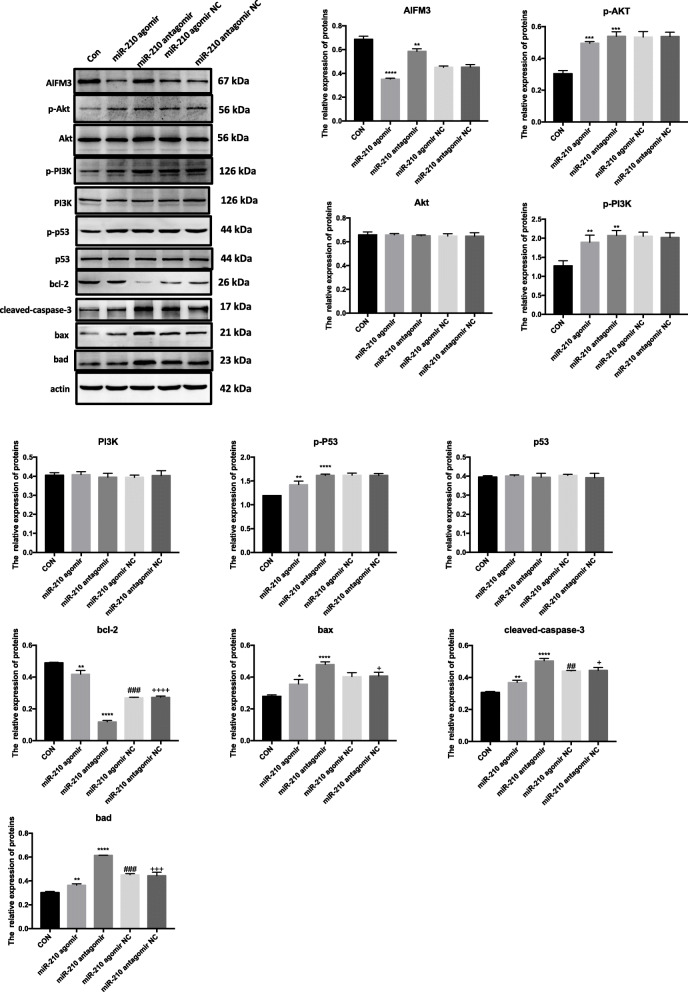
Comparison of the uptake of exosomal miR-210 with the regulatory activity of endogenous miR-210. The protein levels of AIFM3, p-AKT/AKT, p-PI3K/PI3K, p-p53/p53, BAD, BAX, BCL-2, and Cleaved-CASPASE-3 were evaluated by Western blotting in cardiomyocytes treated with overexpressed miR-210. Quantifications are shown in the lower panel. *N* = 3 independent experiments. **p < 0.01, ***p < 0.001, ****p < 0.0001 for miR-210 agomir and miR-210 antagomir vs. con. ^##^*p* < 0.01, ^###^*p* < 0.001 for miR-210 agomir NC vs. miR-210 agomir. ^+^*p* < 0.05, ^+++^*p* < 0.001, ^++++^*p* < 0.0001 for miR-210 antagomir NC vs. miR-210 antagomir

To compare the regulatory activity between endogenous miR-210 and exogenous miR-210, we overexpressed the miR-210 in cardiomyocytes. Western blotting analysis revealed that expression levels of AIFM3, p-AKT, p-PI3K, and p53 were downregulated in cardiomyocytes overexpressing miR-210 versus cardiomyocytes knocked-down for miR-210 (Fig. 8). Also, expression levels of BCL-2 were significantly upregulated, while the levels of Cleaved-CASPASE-3, BAD, and BAX were downregulated (Fig. 8).

## Discussion

Over the past decade, pioneering preclinical research into the use of cell therapy for cardiac regeneration has been completed [19]. However, cell therapy remains challenging for a number of reasons including the selection of cell types, the minimal potency of injected cells, and the limited engraftment and retention of administered cells [19–21]. A recent study demonstrated that although transplanted, cells could not differentiate into cardiac myocytes [22]. In this study, we have identified a mechanism by which the hypoxia-injured MSC “signals” to decrease apoptosis of ischemic cardiomyocytes. We have demonstrated that following MI, exosomes from MSC and their cargo miRNAs were protective for the cardiomyocytes. Notably, the transferred miR-210 downregulates PI3K/Akt expression in the cardiomyocytes, resulting in apoptosis.

Not unexpectedly, exosomes, which have identified paracrine signaling capacities, are being considered as possible therapies for a range of diseases. Consistent with our results, exosomes derived from MSCs prevented ventricular remodeling and reduced scarring in a mouse model of MI [11]. Extending these reports, we explored the possibility that exosome-mediated repair under these conditions relies on the transfer of miRNA. In support of this idea, others found that adipose tissue macrophages from obese mice secrete exosomal-miR-155 to promote glucose intolerance and insulin resistance [23]. miR-105 has been linked to sustained tumor growth [24]. Also, cancers can transfer activated epidermal growth receptor via exosomes to host macrophages and suppress innate antiviral immunity [25]. In this study, we used a miRNA array and found that MSC exosomes were enriched for miR-210. Of relevance to this finding, hypoxia has been reported to increase miR-210 [26]. In addition, it was reported that miR-210 promotes stabilization of carotid plaques via directly targeting the tumor suppressor gene adenomatous polyposis coli [16]. Similarly, increased expression of miR-210 enhanced the protective effects on Hypoxia/reoxygenation-injured endothelial cells [14]. Consistent with this, we observed that the treatment with MSC exosomal-miR-210 improved the survival of cardiomyocytes under the hypoxic condition in vitro and improved heart function in vivo and this was associated with changes in expression of the miR-210 target genes PI3K/Akt and p53. These results are in accordance with several studies suggesting that miR-210 has a protective function in cardiovascular disease through several mechanisms including SOCS1-STAT3-VEGF-C signaling [27]. Consistent with this finding, blocking the activity of nSMase2 lead to a reduction in miR-210 and abrogated any cardio-protective actions.

The regulation of AIFM3 by miR-210 has been reported in cardiomyocytes [28]. Likewise, we found that upregulation of exosomal-miR-210 decreased AIFM3, p-AKT, and p-p53. These effects were eliminated by the knockdown of miR-210. Studies have found that miR-210 has other targets including PLK1, ISCU, Toll-like receptor, and Kruppel-like factor 7 [29–32]. Expanding on this data, we identified AIFM3 as a novel target of miR-210.

The relative contributions of endogenous and exogenous miRNAs in regulating cell responses are controversial. Most studies fail to demonstrate a function for endogenous exosomal miRNAs [33]. We directly overexpressed miR-210 in cardiomyocytes to compare the difference between the endogenous and induced miR-210 [34]. While our results suggest a possible role for endogenous exosomal miRNA, additional exploration of this is required.

This study has a number of limitations. First, the standardization of exosome harvest, handling, and characterization is ongoing [35]. As such, we selected one of several published approaches. While comparison of techniques would be ideal, this was not practical for this study. Second, confocal imaging is not able to distinguish molecular proximity from interaction. Finally, exosomes express cell surface proteins, such as anti-angiogenic thrombospondin-1, that have profound singling effects in the cardiovascular system. Thus, we cannot exclude the possible role of exosome membrane expressed molecules in our assays.

## Conclusions

In conclusion, our study demonstrated the benefits associated with MSC exosomes after MI as well as a novel mechanism responsible for their anti-apoptotic effect via miR-210. Collectively, these results provide new insights into the mechanism of cell therapy and might be leveraged for the treatment of ischemic heart diseases.

## Supplementary information

**Additional file 1: Figure S1.** Exosomes densities and miR-210 levels in different conditions.

**Additional file 2: Figure S2.** Successful construction of AIMF3 promoter report vector.

**Additional file 3: Table S1.** Primer used for PCR.

## Data Availability

All data generated or analyzed in this study are included in this article (and its supplementary information files).

## Abbreviations

MI: Myocardial infarction
MSC: Mesenchymal stem cell
BMSC: Bone mesenchymal stem cell
TEM: Transmission electron micrography
NTA: Nanosight Tracking Analysis
FBS: Fetal bovine serum
PBS: Phosphate-buffered saline
RT-PCR: Reverse transcription-polymerase chain reaction
EF: L Ejection fraction
FS: Fractional shortening
LVIDd: Left ventricular end-diastolic internal diameters
LVIDs: Left ventricular end-systolic internal diameters
