# Supplementary material for: Hypoxia-challenged MSC-derived exosomes deliver miR-210 to attenuate post-infarction cardiac apoptosis
Source: Stem Cell Res Ther. 2020 Jun 8;11:224. doi: 10.1186/s13287-020-01737-0 (PMC7278138; doi:10.1186/s13287-020-01737-0)
Supplement: Supplementary file 1 — Additional file 1: Figure S1. Exosomes densities and miR-210 levels in different conditions. [file 13287_2020_1737_MOESM1_ESM.pdf]

E

GGGGGAGTGCAGACTTTCTCTATCGATAGGTACCGCCACCGCGAAGAGTGGGAC
TTGGATTGGAAAAAGAAACAAGCTTCCAGCTGACTTGCACCCCCGGCCGGGTCCT
GGAGTCCTGCGGGATCCCGTGGAACCTTTGTGAGGTGGGGGTAACTCAGAACCTG
TTTCAGGTTGCTGGAGTTCCTGGGACATGGACGCAAACTCCTCTTGTGGAGTGTG
CAGCAGGGCAATGTTATAACTTTTCTCGGCAGACGTCTCTCCTCGCAGGCCCAAG
AAACTTGTCTTTGAGTTGGAACCACTGGCTCTAAACTGGCCTAGCACATCATCAT
AGGTGGAAGGCATGGTCCCCACCACATCTCCCGCTAGGCCCAAGGAATAAACTC
CTGAACACCCGGAAAGGGAGGGACCCCAGATCGAAAAGCCAGTCTGTTTGTAGG
GTCTCAGTCCCAACAGGTGAGGCCTGAAACCAGGCCGGAAGCAGGTGGGCAGCC
CTGAGACATGGGTGAAACCAGGACTTCTGAAAGGGTTCCCGGGAAGGGAGCTAG
AGGGGCAGTTAGCAACTGAGTTTTATAGACATGCCCTGGTGACAAGGGGGGCTTT
GGGTTCCTCCTCCATTGGAGTTTGCATCAAAGGTCAGGAGCCGGCAGTGGCTGGT
CCTTTCCCATTCTGCTCTTCCACGGGCTCTTTCCTGGGGTCTGGACAAACCGAATG
ATTCCCAGCACCAGTTCAGTATAAACTTCAGGGAGAGTTTCCTGGTGAACCAGGC
CTGCTGTTGGGTAGAATTCGGACAGATTACTTGTAAACAGGGCAGTTACACCTTT
GTCCTGACCCTTCCAAAGTCCACATATTCTAGAAGGCCCACCTATGGCCACAAGG
ACACAATGGCTGTAGTGATCTGTTTACCAGGTAGTCTTGCCTGCCGGTTTGGAGC
CTCAGAGTGATCTGGGGACCTCCCAGGGCCTTACTTATCTTCGGATTTATTAGGGT
GAGGAGTGGGGGAGACCACACAGATGCCCCTAACTGCTGCTTGACCTTAAAGAA
ACCTTGGCCCCCAGTCCTATCTTCTGTGGGGCGTGACCTCGGCACTGCACAGCCA
GCCTGAGGGGTGGGGGAACTTATGGCTGCTGGGCATGGGGCAGAGGCAGAGCTG
TGCGGTTGGGTTGGTTTCCCTCTTTCCTCAACCCCATCAATCCTGGGCTGGCTGCC
CGCTCTTTTCCCCACAGAGCTGGGTTTCCCTGGGGACCAGTGGCTTGGCCTAGCCT
CACACCCACAGCCCCCTCCTGGCCACAACTCACGCTAGAAGGAGGGCTGGACCCT
CCTGGACCCTGGCCCTCCCTGCCCTGCTGACCAAGGTCCAGTAGTCTCTCTCCATT
GCTGGGCTGGGAGCAAGGAGCAGTGACTCTCACGGAAGGAGGTAGGTGACTAGA
GGTTGCTAGTCAGGGTAGTGGCCAAGGCTAGGGTGGGAATCTTGTCACTCTTCTT
GTCACTAGGGTGGAAGAAGAAGGATTCTGGGGACTCTGGTTGTCAGGGTTTGCTA
TAGACTTGGTGATCTTACCTAGGGGAATGGAGAGTCCCAGAGTCCCCAGACTTGT
GGGACTTAGAAAGGGGAGTCTGGGATTAGGTCTCTGCAGTCACAGGGTTATGGG
CTGTGGCAGACACTAGACTACAGAGGGTCTCCAACAATGCATGCAGTAAGGGGA
GTATGAGGTCCCCATGCCAGGAAGCCTCAAAGGGAGACAGATACTATCAAGAGT
ACCCTACAAACGCGTTCTGAGCTCCCCATGTAAGACTCTTCTAGGATCCTCCAAG
GATGCTTCTGACTCTACCTGCATCTGCATCGGTGCTGCATCTGCATCGGTGCTACG
ACCCAATGTCCCATGTCCCCTGCAGGTTCTAAAGCTGTCTAGCTGGCATGGCAGC
TCCAGCGTCTCTGAGCGCCACAGGGGGTCCAGTCCTACAGGGCTCATCCTAGACC
TCGGACAGCTGGACAGCCTGCCATCCGTGCTCCCACCAGCCGGGCTAGATCCAGG
CCATTCAACCCTCGAGATCTGCGATCTAAGTAAGCTTGGCATTCCGGTACTGTTG
GTAAAGCCACCATGGAAGACGCCAAAAACATAAAGAAAGGCCCGGCGCCATTCT
ATCCG

F

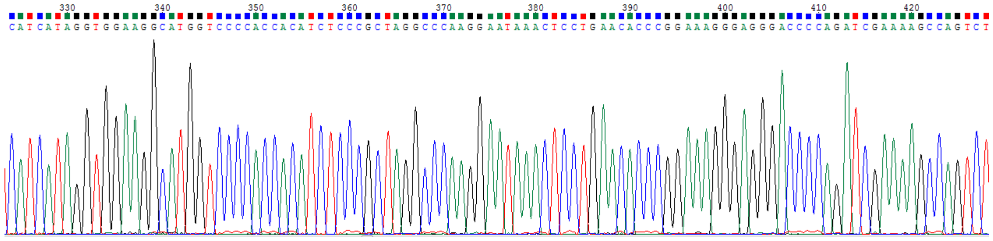

Figure S2.
Successful construction of AIMF3 promoter report vector
(A) PGL3 vector map
(B) Amplification results of gene AIFM3 promoter region
(C) Colony PCR verification results
(D) Verification of overexpression plasmid PGL3-AIFM3 promoter region digestion
(E) Plasmid PGL3-AIFM3 promoter region
(F) Sequencing results
