# Supplementary material for: Hypoxia-challenged MSC-derived exosomes deliver miR-210 to attenuate post-infarction cardiac apoptosis
Source: Stem Cell Res Ther. 2020 Jun 8;11:224. doi: 10.1186/s13287-020-01737-0 (PMC7278138; doi:10.1186/s13287-020-01737-0)
Supplement: Supplementary file 2 — Additional file 2: Figure S2. Successful construction of AIMF3 promoter report vector. [file 13287_2020_1737_MOESM2_ESM.pdf]

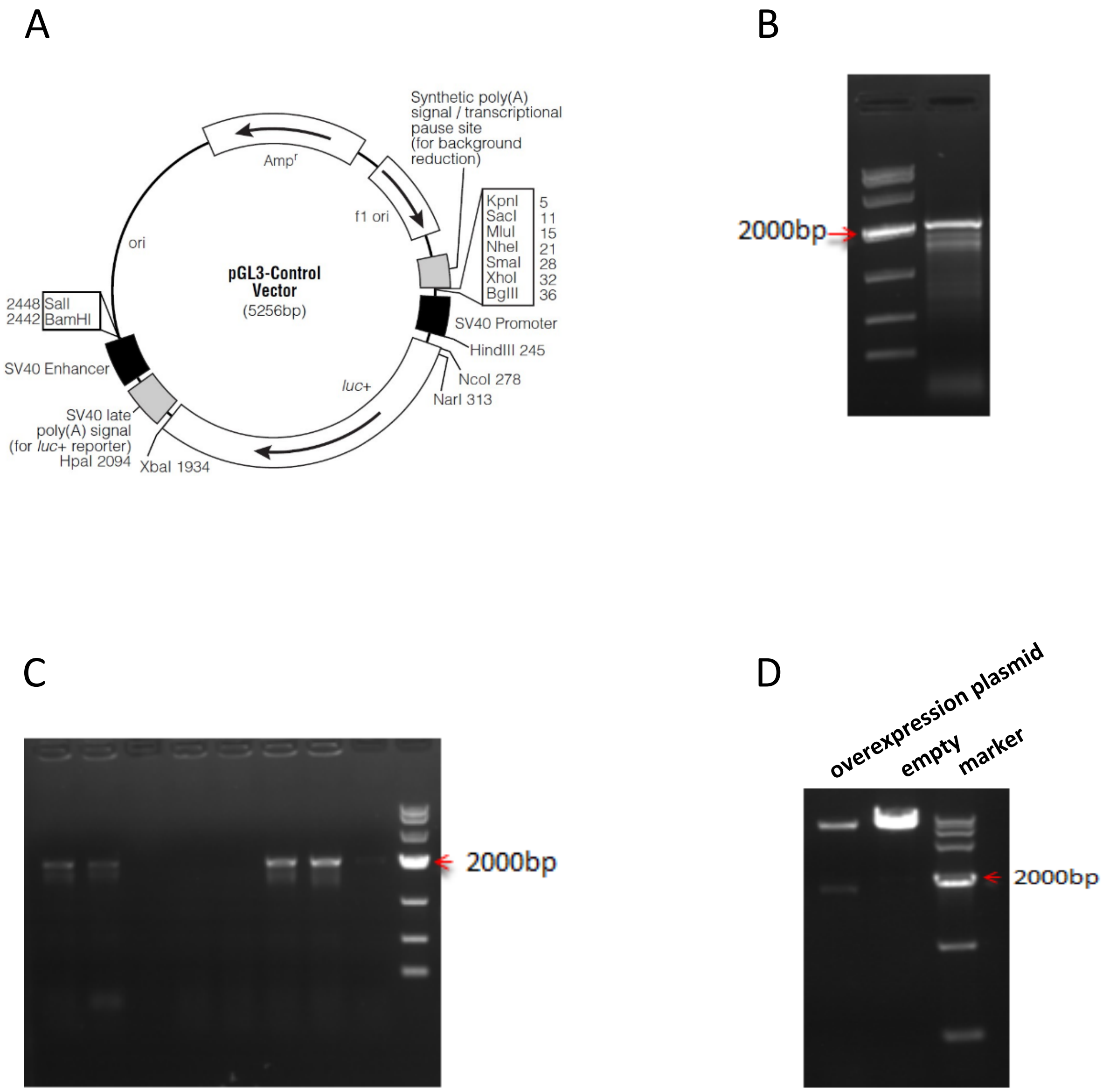
A
Synthetic poly(A) signal / transcriptional pause site (for background reduction)
Amp^r
f1 ori
ori
KpnI 5
SacI 11
MluI 15
NheI 21
SmaI 28
XhoI 32
BglII 36
pGL3-Control Vector
(5256bp)
2448 SalI
2442 BamHI
SV40 Promoter
HindIII 245
SV40 Enhancer
NcoI 278
NarI 313
luc+
SV40 late poly(A) signal (for luc+ reporter)
HpaI 2094
XbaI 1934
B
2000bp
C
2000bp
D
overexpression plasmid
empty
marker
2000bp

E

GGGGGAGTGCAGACTTTCTCTATCGATAGGTACCGCCACCGCGAAGAGTGGGAC
TTGGATTGGAAAAAGAAACAAGCTTCCAGCTGACTTGCACCCCCGGCCGGGTCCT
GGAGTCCTGCGGGATCCCGTGGAACCTTTGTGAGGTGGGGGTAACTCAGAACCTG
TTTCAGGTTGCTGGAGTTCCTGGGACATGGACGCAAACTCCTCTTGTGGAGTGTG
CAGCAGGGCAATGTTATAACTTTTCTCGGCAGACGTCTCTCCTCGCAGGCCCAAG
AAACTTGTCTTTGAGTTGGAACCACTGGCTCTAAACTGGCCTAGCACATCATCAT
AGGTGGAAGGCATGGTCCCCACCACATCTCCCGCTAGGCCCAAGGAATAAACTC
CTGAACACCCGGAAAGGGAGGGACCCCAGATCGAAAAGCCAGTCTGTTTGTAGG
GTCTCAGTCCCAACAGGTGAGGCCTGAAACCAGGCCGGAAGCAGGTGGGCAGCC
CTGAGACATGGGTGAAACCAGGACTTCTGAAAGGGTTCCCGGGAAGGGAGCTAG
AGGGGCAGTTAGCAACTGAGTTTTATAGACATGCCCTGGTGACAAGGGGGGCTTT
GGGTTCCTCCTCCATTGGAGTTTGCATCAAAGGTCAGGAGCCGGCAGTGGCTGGT
CCTTTCCCATTCTGCTCTTCCACGGGCTCTTTCCTGGGGTCTGGACAAACCGAATG
ATTCCCAGCACCAGTTCAGTATAAACTTCAGGGAGAGTTTCCTGGTGAACCAGGC
CTGCTGTTGGGTAGAATTCGGACAGATTACTTGTAAACAGGGCAGTTACACCTTT
GTCCTGACCCTTCCAAAGTCCACATATTCTAGAAGGCCCACCTATGGCCACAAGG
ACACAATGGCTGTAGTGATCTGTTTACCAGGTAGTCTTGCCTGCCGGTTTGGAGC
CTCAGAGTGATCTGGGGACCTCCCAGGGCCTTACTTATCTTCGGATTTATTAGGGT
GAGGAGTGGGGGAGACCACACAGATGCCCCTAACTGCTGCTTGACCTTAAAGAA
ACCTTGGCCCCCAGTCCTATCTTCTGTGGGGCGTGACCTCGGCACTGCACAGCCA
GCCTGAGGGGTGGGGGAACTTATGGCTGCTGGGCATGGGGCAGAGGCAGAGCTG
TGCGGTTGGGTTGGTTTCCCTCTTTCCTCAACCCCATCAATCCTGGGCTGGCTGCC
CGCTCTTTTCCCCACAGAGCTGGGTTTCCCTGGGGACCAGTGGCTTGGCCTAGCCT
CACACCCACAGCCCCCTCCTGGCCACAACTCACGCTAGAAGGAGGGCTGGACCCT
CCTGGACCCTGGCCCTCCCTGCCCTGCTGACCAAGGTCCAGTAGTCTCTCTCCATT
GCTGGGCTGGGAGCAAGGAGCAGTGACTCTCACGGAAGGAGGTAGGTGACTAGA
GGTTGCTAGTCAGGGTAGTGGCCAAGGCTAGGGTGGGAATCTTGTCACTCTTCTT
GTCACTAGGGTGGAAGAAGAAGGATTCTGGGGACTCTGGTTGTCAGGGTTTGCTA
TAGACTTGGTGATCTTACCTAGGGGAATGGAGAGTCCCAGAGTCCCCAGACTTGT
GGGACTTAGAAAGGGGAGTCTGGGATTAGGTCTCTGCAGTCACAGGGTTATGGG
CTGTGGCAGACACTAGACTACAGAGGGTCTCCAACAATGCATGCAGTAAGGGGA
GTATGAGGTCCCCATGCCAGGAAGCCTCAAAGGGAGACAGATACTATCAAGAGT
ACCCTACAAACGCGTTCTGAGCTCCCCATGTAAGACTCTTCTAGGATCCTCCAAG
GATGCTTCTGACTCTACCTGCATCTGCATCGGTGCTGCATCTGCATCGGTGCTACG
ACCCAATGTCCCATGTCCCCTGCAGGTTCTAAAGCTGTCTAGCTGGCATGGCAGC
TCCAGCGTCTCTGAGCGCCACAGGGGGTCCAGTCCTACAGGGCTCATCCTAGACC
TCGGACAGCTGGACAGCCTGCCATCCGTGCTCCCACCAGCCGGGCTAGATCCAGG
CCATTCAACCCTCGAGATCTGCGATCTAAGTAAGCTTGGCATTCCGGTACTGTTG
GTAAAGCCACCATGGAAGACGCCAAAAACATAAAGAAAGGCCCGGCGCCATTCT
ATCCG

F

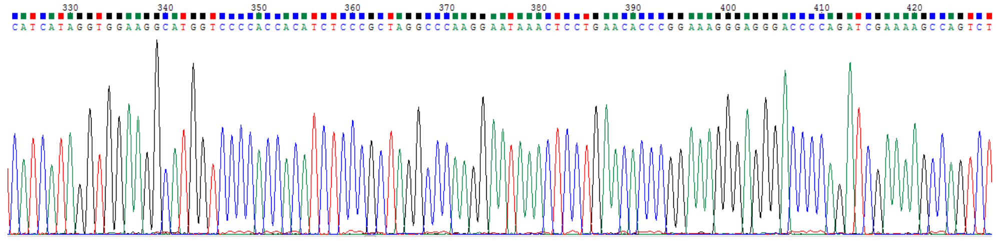

Figure S2.
Successful construction of AIMF3 promoter report vector
(A) PGL3 vector map
(B) Amplification results of gene AIFM3 promoter region
(C) Colony PCR verification results
(D) Verification of overexpression plasmid PGL3-AIFM3 promoter region digestion
(E) Plasmid PGL3-AIFM3 promoter region
(F) Sequencing results
