# Supplementary material for: Hypoxia-challenged MSC-derived exosomes deliver miR-210 to attenuate post-infarction cardiac apoptosis
Source: Stem Cell Res Ther. 2020 Jun 8;11:224. doi: 10.1186/s13287-020-01737-0 (PMC7278138; doi:10.1186/s13287-020-01737-0)
Supplement: Supplementary file 3 — Additional file 3: Table S1. Primer used for PCR. [file 13287_2020_1737_MOESM3_ESM.pdf]

Table S1. Primer used for PCR

AIFM3 promoter region	forward: 5’-CGGGGTACCGCCACCGCGAAGAGTGGGACTTGGATT-3’	2000bp
	reverse: 5’-CCGCTCGAGGGTTGAATGGCCTGGATCTAG-3’	
